# Identification of TAZ-Dependent Breast Cancer Vulnerabilities Using a Chemical Genomics Screening Approach

**DOI:** 10.3389/fcell.2021.673374

**Published:** 2021-06-15

**Authors:** He Shen, Yanmin Chen, Yin Wan, Tao Liu, Jianmin Wang, Yali Zhang, Lei Wei, Qiang Hu, Bo Xu, Mikhail Chernov, Costa Frangou, Jianmin Zhang

**Affiliations:** ^1^Department of Cancer Genetics and Genomics, Roswell Park Comprehensive Cancer Center, Buffalo, NY, United States; ^2^Department of Biostatistics and Bioinformatics, Roswell Park Comprehensive Cancer Center, Buffalo, NY, United States; ^3^Department of Pathology, Roswell Park Comprehensive Cancer Center, Buffalo, NY, United States; ^4^Department of Cell Stress Biology, Roswell Park Comprehensive Cancer Center, Buffalo, NY, United States; ^5^Molecular and Integrative Physiology Department, Harvard T.H. Chan School of Public Health, Boston, MA, United States

**Keywords:** Hippo pathway, TAZ, breast cancer, stem cells, small molecule, Cyclin D, CDK4/6

## Abstract

Breast cancer stem cells (BCSCs) represent a subpopulation of tumor cells that can self-renew and generate tumor heterogeneity. Targeting BCSCs may ameliorate therapy resistance, tumor growth, and metastatic progression. However, the origin and molecular mechanisms underlying their cellular properties are poorly understood. The transcriptional coactivator with PDZ-binding motif (TAZ) promotes mammary stem/progenitor cell (MaSC) expansion and maintenance but also confers stem-like traits to differentiated tumor cells. Here, we describe the rapid generation of experimentally induced BCSCs by TAZ-mediated reprogramming of human mammary epithelial cells, hence allowing for the direct analysis of BCSC phenotypes. Specifically, we establish genetically well-defined TAZ-dependent (TAZ_DEP_) and -independent (TAZ_IND_) cell lines with cancer stem cell (CSC) traits, such as self-renewal, variable resistance to chemotherapeutic agents, and tumor seeding potential. TAZ_DEP_ cells were associated with the epithelial to mesenchymal transition, embryonic, and MaSC signature genes. In contrast, TAZ_IND_ cells were characterized by a neuroendocrine transdifferentiation transcriptional program associated with Polycomb repressive complex 2 (PRC2). Mechanistically, we identify Cyclin D1 (CCND1) as a critical downstream effector for TAZ-driven tumorigenesis. Overall, our results reveal a critical TAZ-CCND1-CDK4/CDK6 signaling axis, suggesting novel therapeutic approaches to eliminate both BCSCs and therapy-resistant cancer cells.

## Introduction

The genomic characterization of thousands of primary breast tumors has revealed considerable spatiotemporal heterogeneity within the tumors from individual patients, presenting a formidable challenge when it comes to diagnosis, prognosis, and developing effective breast cancer (BC) therapeutics. Two prevailing models have been proposed to account for tumor heterogeneity. In the clonal evolution model, stochastic mutations in individual tumor cells serve as a platform for the adaptation and natural selection of the fittest variants. Cancer clone epigenetic and/or genetic diversification within tissue microenvironments confers phenotypic, behavioral, and functional differences among BC cells (Meacham and Morrison, 2013). In contrast, the cancer stem cell (CSC) model posits that a subset of cells in tumors can both self-renew and differentiate into diverse cancer cell hierarchies, which play a decisive role in tumor growth, progression, recurrence, and treatment resistance.

The importance of targeting CSCs [also known as tumor-initiating cells (TICs)] derives from the multiple clinical and experimental observations showing that CSCs have increased tumor seeding potential and are resistant to chemotherapeutic agents and ionizing radiation (Brooks et al., 2015; Vlashi and Pajonk, 2015). Extensive research has focused on discovering and characterizing CSCs at the origin of different tumors. Despite these efforts, the definition of CSCs remains largely operational and based on the functional assays that monitor their self-renewal and tumorigenic properties (i.e., the formation of tumor spheres *in vitro* and heterogeneous tumors at limiting dilutions *in vivo*). Nonetheless, CSC-enriched cancer cell populations can be isolated using cell-surface marker profiles. For instance, BC stem cells (BCSCs) were first identified as a CD24^–/low^/CD44^+^ population with an enhanced ability to initiate tumor growth when xenografted into immunocompromised mice (Al-Hajj et al., 2003). Subsequent studies have identified additional markers, such as Aldeflour, that measure the ALDG (aldehyde dehydrogenase) activity in mammary stem/progenitor cells (MaSCs) (Ginestier et al., 2007). Whether these markers are universally expressed in tumors, identify the same BC cell population, or correspond to therapeutic response remains unclear.

The ability of cells to adopt different and reversible identities is a phenomenon known as cellular plasticity. Dynamic interconversions between transitional epithelial and mesenchymal states predicate the intrinsic plasticity observed in mammary epithelial cells (MECs) in response to exogenous stimuli and microenvironment factors. Under such conditions, cellular plasticity serves as a tissue adaptation mechanism, but it can also predispose cells to malignant transformation and tumorigenesis. For example, epithelial-mesenchymal transition (EMT), where cells with an epithelial phenotype transition to display a mesenchymal phenotype while maintaining the capacity to reassume their epithelial state, is the best-known case of tumor cell plasticity (Kalluri and Weinberg, 2009; Chaffer et al., 2016; Brabletz et al., 2018). Notably, when EMT is aberrantly activated in cancer, cells gain attributes of stem cells that contribute to self-renewal capabilities and can differentiate to all cell types represented in the tumor (Mani et al., 2008). Although many studies have defined the association between EMT induction and acquisition of stemness, few have addressed the mechanisms by which EMT directly induces BCSCs and link these two cellular states.

Several pathways have been implicated in BCSC expansion and maintenance regulation, including Hedgehog, Hippo, TGF-β, Notch, and Wnt/β-catenin (Yousefnia et al., 2020). Hippo signaling is an evolutionarily conserved pathway that controls tissue and organ size by regulating cell proliferation, apoptosis, and MaSC self-renewal (Zheng and Pan, 2019). Hippo signaling involves a highly conserved core kinase cascade, including MST1/2, LATS1/2, and the transcriptional coactivators YAP (Yes-associated protein) and TAZ (WW domain-containing transcription regulator protein), which are downstream effectors of the pathway (Moroishi et al., 2015; Zanconato et al., 2016). Recent studies have highlighted YAP/TAZ’s role in regulating MEC plasticity. For instance, the overexpression of TAZ can induce EMT (Chan et al., 2008; Lei et al., 2008; Yang et al., 2012). Furthermore, the transient expression of exogenous TAZ in primary differentiated mouse MECs can induce the conversion of a tissue-specific stem or progenitor cell state (Panciera et al., 2016). Similarly, the hyperactivation of YAP/TAZ gives non-BCSCs the ability to remain undifferentiated, self-renew, and metastasize (Cordenonsi et al., 2011; Kim et al., 2015).

Understanding the molecular mechanisms that underlie BCSC properties has been greatly hindered because of the difficulty in isolating rare and heterogeneous CSCs from bulk tumor tissues and propagating the cultures of the BCSCs *in vitro*. Here, we describe an experimental workflow that allows for the rapid isolation of these cells from mammary tumors with defined genotypes. Using this approach, we identify a TAZ-CCND1-CDK4/CDK6 signaling axis that is involved in BCSC self-renewal and propose that CDK4/6 inhibitors can serve as a potential therapeutic drug to target TAZ dependency in these cells.

## Results

### Isolation of Tumor-Initiating Cells From Human BC Xenografts

**FIGURE 1:**
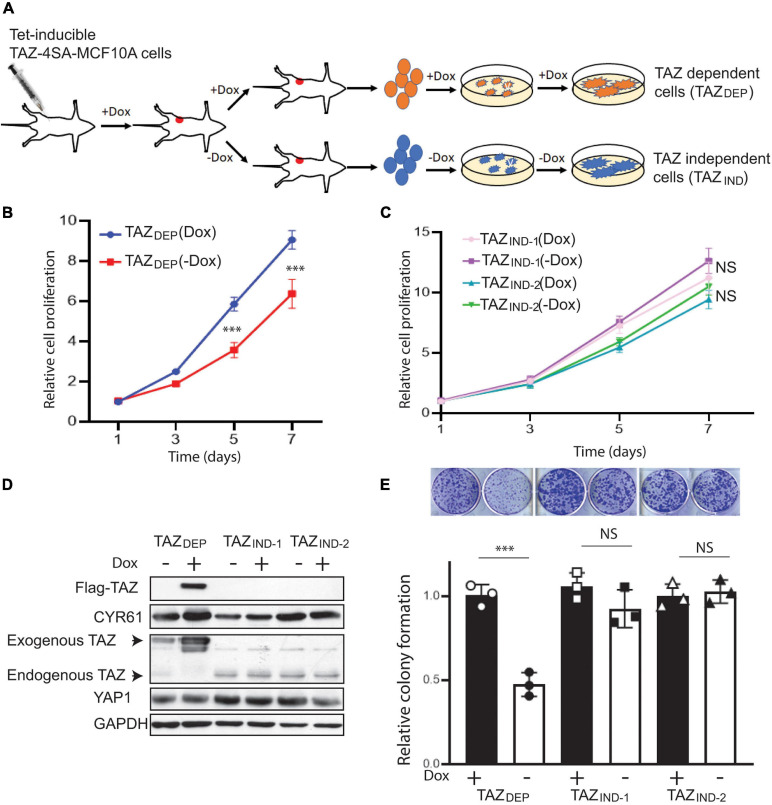
Isolation of TAZ_DEP_ and TAZ_IND_ tumor-derived isogenic cells. **(A)** Schematic description of the preparation of TAZ_DEP_ and TAZ_IND_ tumor-derived isogenic cells. **(B)** Cell proliferation assay of TAZ_DEP_ cells in response with or without dox treatment (2 μg/ml). Data are shown as the mean ± SD. Unpaired two-tailed Student’s *t*-test: ^∗∗∗^*p* < 0.001. **(C)** Cell proliferation assay of TAZ_IND_ cells in response with or without dox treatment (2 μg/ml). Data are shown as the mean ± SD. Unpaired two-tailed Student’s *t*-test: NS = not significant. **(D)** Exogenous (Flag-TAZ) and endogenous TAZ, CTGF, and YAP expression detected by immunoblotting. GAPDH was used as a loading control. **(E)** Representative images and quantification of colony formation of TAZ_DEP_ and TAZ_IND_ cells in response to or without dox treatment. Data are shown as the mean ± SD. Unpaired two-tailed Student’s *t*-test: ^∗∗∗^*p* < 0.001; NS = not significant.

To understand BCSC phenotypes and identify the pathways involved in their genesis and maintenance, we set out to establish a robust platform for enriching TICs from human tumor xenografts. We previously described a transplant model of BC in which constitutively active TAZ expression is controlled by the reverse transactivator system in a dox-dependent manner (Shen et al., 2019). Upon dox removal, most mammary tumors regressed; however, in a subcohort of mice, we also observed tumor growth that occurred independent of TAZ expression (Figure 1A). We reasoned that these mammary tumors contained TICs with stem markers and functional stem cell traits from the different oncogenic drivers required for tumor growth and progression.

We harvested TAZ-dependent (12 mice) and -independent mammary tumors (5 mice). Thereafter, for TICs, we procured samples from bulk tumor cells using mammosphere growth conditions, which rely on the fact that cells with stemness features preferentially respond to growth factors and grow in suspension as clonal non-adherent spherical clusters (Fillmore and Kuperwasser, 2008). Herein, we describe three genetically characterized cell lines derived from MCF10A-TAZ mammary tumors (Supplementary Table 1): TAZ-dependent cells (hereafter denoted as TAZ_DEP_) and two TAZ-independent cell lines (TAZ_IND_). As shown in Figure 1B, TAZ_DEP_ and TAZ_IND_ cell proliferation rates were similar in the 2D culture. However, we observed a dramatic decrease in cell proliferation, viability, and long-term colony formation capacity for TAZ_DEP_ cells upon withdrawal of dox (Figures 1B–E and Supplementary Figures 1A,B). As expected, we did not detect high and sustainable TAZ expression in TAZ_IND_ cells (Figure 1D) because of inactivation or silencing of the transgene cassette *in vivo*.

### Characterization of TAZ_DEP_ and TAZ_IND_ Tumor Cells

**FIGURE 2:**
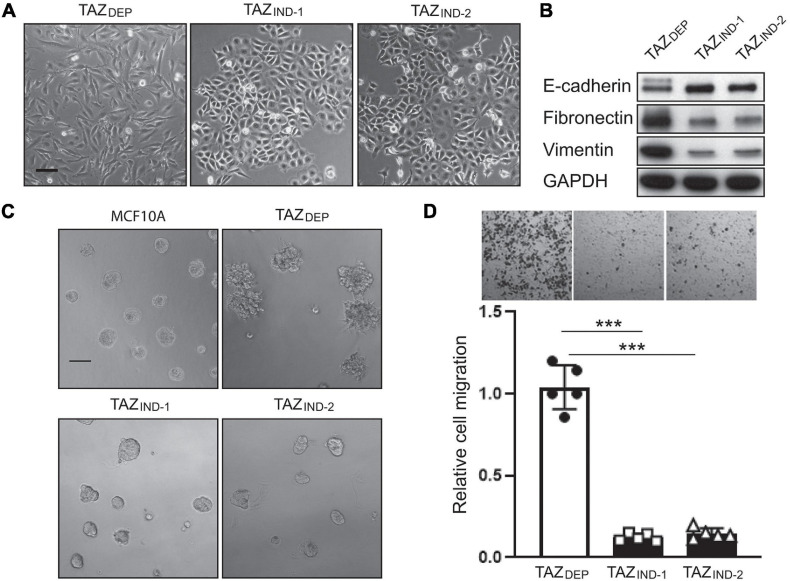
TAZ_DEP_ cells undergo EMT. **(A)** Representative images of TAZ_DEP_ and TAZ_IND_ cell morphology in a 2D culture. TAZ_DEP_ cells grown in the presence of 2 μg/ml dox. Scale bar = 50 μm. **(B)** Immunoblotting detection of E-cadherin, fibronectin, and vimentin in TAZ_DEP_ and TAZ_IND_ cells. GAPDH was used as a loading control. **(C)** Representative images of MCF10A, TAZ_DEP_, and TAZ_IND_ cells grown in a 3D culture. Scale bar = 100 μm. **(D)** Representative images and quantification of TAZ_DEP_ and TAZ_IND_ cell migration. Data are shown as the mean ± SD. Unpaired two-tailed Student’s *t*-test: ***p < 0.001.

EMT is relevant to the acquisition and maintenance of stem cell-like characteristics and is sufficient for endowing differentiated MECs and BC cells with stem cell properties (Mani et al., 2008). Moreover, EMT can generate a spectrum of cellular states displaying mixed epithelial and mesenchymal features between these two extremes *in vitro* and *in vivo* (Celia-Terrassa et al., 2012; Kroger et al., 2019). We (among other studies) have previously shown that TAZ activation induced EMT in MCF10A cells (Lei et al., 2008; Li et al., 2015). Consistent with these findings, TAZ_DEP_ cells displayed mesenchymal morphologies (Figure 2A), whereas TAZ_IND_ cells maintained the cobblestone morphology characteristic of epithelial cells. To corroborate the observed changes in morphology, we examined changes in the expression of canonical markers of the epithelial and mesenchymal states. TAZ_DEP_ cells were associated with decreased E-cadherin protein expression and increased expression of mesenchymal markers such as fibronectin and vimentin, respectively (Figure 2B). The mesenchymal phenotype was partially reversed by the withdrawal of dox from TAZ_DEP_ cells, suggesting TAZ regulates cellular plasticity (Supplementary Figures 1C,E).

3D culture models allow for phenotypic discrimination between non-malignant and malignant MEC clones because they can recapitulate organotypic growth. For instance, transformed cells adopt various colony morphologies, including a loss of tissue polarity, a disorganized architecture, and the failure to arrest growth (De Angelis et al., 2019). With this in mind, we investigated non-malignant and tumor-derived mammary cell phenotypes and growth in a 3D context. As expected, MCF10A cells organized into polarized colonies with many of the morphological features of mammary acini (Figure 2C). TAZ_DEP_ cells formed enlarged acini with invasive (stellate) structures (Figure 2C). Dox withdrawal inhibited their growth in 3D culture (Supplementary Figure 1D). In contrast, TAZ_IND_ cells formed smaller round acini (Figure 2C). Consistent with these observations, cell migration potential—-as assessed by Boyden chamber assays—-was reduced in TAZ_DEP_ vs. TAZ_IND_ cells (Figure 2D). Together, these results reaffirm previous work that constitutive TAZ expression promotes an EMT program that enables TAZ_DEP_ cells to assume a mesenchymal cell phenotype, including enhanced migratory capacity and invasiveness (Chan et al., 2008). However, this occurs independently of BC cell proliferation.

### Maintenance of BCSC Properties and Tumorigenic Potential

**FIGURE 3:**
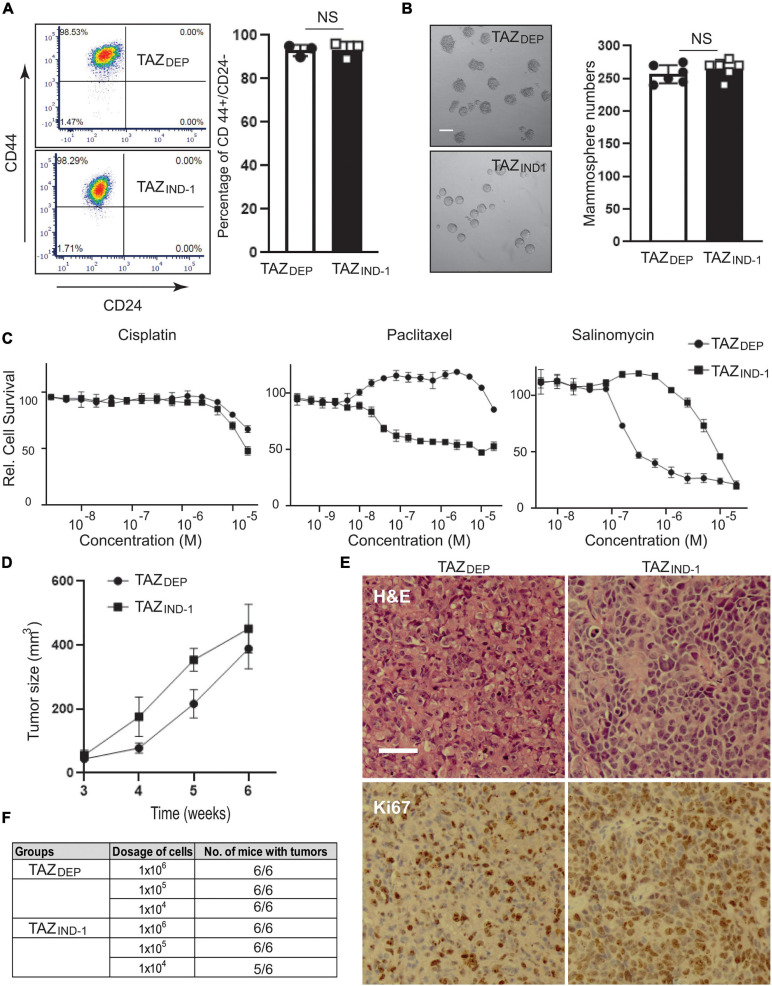
Conservation and BCSC features and tumor formation potential. **(A)** Representative images and quantification of CD44^*high*^/CD24^*low*^ cell population of TAZ_DEP_ and TAZI_ND_ cells by FACS analysis. Data are shown as the mean ± SD. Unpaired two-tailed Student’s *t*-test: NS = not significant. **(B)** Representative images and quantification of mammosphere formation of TAZ_DEP_ and TAZ_IND_ cells. Scale bar = 100 μm. Data are shown as the mean ± SD. Unpaired two-tailed Student’s *t*-test: NS = not significant. **(C)** Cell survival was tested in response to the various concentrations of Cisplatin, Paclitaxel, and Salinomycin for TAZ_DEP_ and TAZ_IND_ cells. TAZ_DEP_ cells grown in the presence of 2 μg/ml dox. **(D)** 1 × 10^6^ TAZ_DEP_ or TAZ_IND_ cells were injected into mammary gland fat pads of SCID mice (*n* = 6). TAZ_DEP_ cells injected into mice were fed with dox-containing chow (Bio-serv, NJ). Tumor growth rates were measured weekly by caliper. **(E)** H&E and Ki67 immunohistochemical (IHC) staining for TAZ_DEP_ -or TAZ_IND_ tumors. Scale bar = 50 μm. **(F)** Serial diluted (1 × 10^6^, 1 × 10^5^, 1 × 10^4^) TAZ_DEP_ or TAZ_IND_ cells were injected into the mammary fat pads of SCID mice (*n* = 6). Tumor formation capability was recorded.

Although BC cell lines provide useful information about cancer biology, their adaptation to the *in vitro* environment and artificial selection pressures in tissue cultures result in biological properties that differ in essential ways from *de novo* tumor cells. BCSC phenotypes may be unstable, resulting in phenotypic reversion of cell surface markers and switching of the CSC phenotype (Visvader and Lindeman, 2012). In addition, most tumors and cell lines possess their own unique ratios of BCSC markers and populations. To investigate this possibility and gain insights into the nature of our experimentally derived BCSCs, we used multiple methods to identify BCSCs *in vitro* and *in vivo*. As shown in Figure 3A, a FACS analysis readily revealed a CD44^high^/CD24^low^ subpopulation in both TAZ_DEP_ and TAZ_IND_ cells (>95%) compared with MCF10A cells (CD44^low^/CD24^low^). Correspondingly, both TAZ_DEP_ and TAZ_IND_ cells were endowed with long-term self-renewal capacity, as measured by mammosphere assays (Figure 3B).

In addition to self-renewal, another characteristic of CSCs is their capacity to resist chemotherapy (Lai et al., 2011). Therefore, we treated MECs with two widely used BC chemotherapeutic drugs, Cisplatin and Paclitaxel. TAZ_DEP_ and TAZ_IND_ cells were more resistant (∼20-fold) than the control MCF10A cells to these drugs (data not shown). As shown in Figure 3C, TAZ_DEP_ cells are more sensitive to Paclitaxel treatment than TAZ_IND_ cells. Salinomycin, an antibacterial and coccidiostat ionophore, has been shown to efficiently suppress BCSC survival (Gupta et al., 2009; Zhou et al., 2013). Strikingly, TAZ_DEP_ cells are 100-fold more sensitive to Salinomycin treatment than TAZ_IND_ cells (Figure 3C), which is consistent with monovalent cation ionophores’ potent and cytostatic effects against EMT-like cells and therapy-resistant cancer cells (Vanneste et al., 2019).

Anchorage-independent growth is one of the hallmarks of cancer. Thus, we examined the effect of TAZ-mediated tumorigenic activity using anchorage-independent growth conditions as an *in vitro* readout of the malignant transformation of MECs. Specifically, we grew isogenic cell lines in soft agar for 10 days and quantified the number of colonies and size of the colonies formed. Both TAZ_DEP_ and TAZ_IND_ cells displayed similar growth properties in soft agar (Supplementary Figure 1F). Next, to confirm tumor formation potential *in vivo*, we injected TAZ_DEP_ or TAZ_IND_ cells into the mammary fat pad of Prkdc**^*scid*^** (SCID) mice. TAZ_DEP_ and TAZ_IND_ cells generated palpable tumors with similar penetrance and growth kinetics (Figure 3D). Histological analysis indicated that both TAZ_DEP_ and TAZ_IND_ cells formed a high-grade carcinoma with high proliferation potential (Figure 3E). However, TAZ_IND_ cells displayed neuroendocrine features, suggesting they may have undergone dedifferentiation (Figure 3E).

Finally, to better assess tumor-initiation capacity, we performed orthotopic transplantation of TAZ_DEP_ and TAZI_ND_ cells in limiting dilution assays (Figure 3F). Our results show that our isogenic cell lines possessed > 30-fold higher tumor-initiating potential than bulk tumor cells. Furthermore, our data suggest that both TAZ_DEP_ and TAZ_IND_ cells display stable and heritable BCSC markers and functional characteristics, including the ability to form long-term tumors *in vivo*.

### Transcriptome Analysis of TAZ_DEP_ and TAZ_IND_ Transformed MECs

**FIGURE 4:**
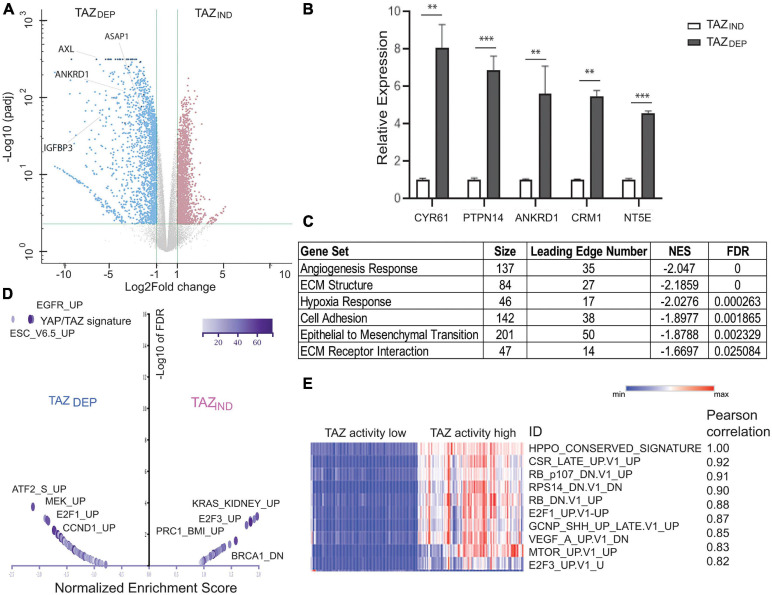
Gene expression profiling for TAZ_DEP_ and TAZ_IND_ cells. **(A)** Volcano plot shows significant gene expression alterations between TAZ_DEP_ and TAZ_IND_ cells. **(B)** qRT-PCR experiment detection of canonical TAZ target expression in TAZ_DEP_ and TAZ_IND_ cells. Relative expression was normalized by GAPDH expression. Unpaired two-tailed Student’s *t*-test: ^∗∗^*p* < 0.01; ^∗∗∗^*p* < 0.001. **(C)** Over-representation analysis (ORA) using pathway annotations. **(D)** Volcano plot summarizes the normalized enrichment score (NES) for MSigDB oncogenic signature sets with direction for TAZ_DEP_ vs. TAZ_IND_ cells. **(E)** Correlation analysis of TAZ_DEP_ and TAZ_IND_ modules using TCGA Breast Invasive Carcinoma patient (RNASeqv2 RSEM) gene expression data. Top 10 pairwise Pearson correlations between TAZ activation and oncogenic gene signatures are summarized.

Elucidation of the pathways that regulate the survival of BCSCs is important for the development of novel therapies. To gain a broader perspective of the underlying biological processes used by TAZ_DEP_ and TAZ_IND_ cells for the maintenance of their BCSC phenotypes, we performed an RNA-seq and over-representation analysis (ORA) using pathway annotations and GO term datasets (Jiao et al., 2012). We identified 1,854 significantly upregulated and 1,937 downregulated genes (twofold change, FDR ≤ 5%; Figure 4A and Supplementary Table 2). qRT-PCR analysis confirmed canonical TAZ target expression (Wang et al., 2018) highly expressed in TAZ_DEP_ cells (Figure 4B). TAZ_IND_ cells were enriched for MaSC and mammary cell signatures (Supplementary Figure 2). TAZ_DEP_ cells were enriched for cell-regulatory and growth processes, including epithelial cell proliferation, cell adhesion, cell-surface proteins, extracellular matrix (ECM) structure proteins, and the production of ECM-degrading enzymes. ECM cleavage and remodeling can promote cell movement, and profoundly influence the directed migration of BC cells. Hence, the deregulation of these biological processes may account for the different migration and invasive properties of TAZ_DEP_ vs. TAZ_IND_ cells.

As a complementary approach to ORA, we performed a gene set enrichment analysis (GSEA) (Subramanian et al., 2005). To expand our analysis beyond pathways, we also included MSigDB gene sets derived from different experiments, each of which represents genes whose expression is altered in response to a perturbation in a known cancer-associated gene (Supplementary Figure 2; Liberzon, 2014). YAP/TAZ target genes and EMT signatures were upregulated in TAZ_DEP_ cells (Figures 4C,D). Similarly, the Cyclin D (CCND1), RB/E2F, epidermal growth factor receptor (EGFR), and MAPK/ERK pathway signatures were also upregulated in TAZ_DEP_ cells (Figure 4D). Conversely, several PRC2 complex target signatures and neuron progenitor features were preferentially enriched in TAZ_IND_ cells (Figure 4C and Supplementary Figures 2, 3).

In order to determine the generality of our findings, we performed a correlation analysis between TAZ transcriptional activity and associated oncogenic pathways/signatures using gene expression data from TCGA BC patients (Ciriello et al., 2015). The top 10 most connected modules are dominated by genetic perturbations of RB and E2F genes, thereby supporting our approach for identifying biologically meaningful relationships (Figure 4E). Collectively, these results suggest that TAZ_DEP_ and TAZ_IND_ cells’ phenotypic characteristics are manifested because they rely on different biological processes that contribute to their unique proliferation, survival, self-renewal, and differentiation properties.

### A Small Molecule Library Screen Identifies Specific Vulnerabilities in TAZ_DEP_ and TAZ_IND_ Cells

Molecular alterations that confer phenotypic advantages to tumors can also expose specific genetic vulnerabilities (Lee et al., 2018). For example, cancers harboring translocations that form fusion transcripts, such as BCR-ABL, or mutations, such as BRAF or EGFR, depend on these gene products’ activity for tumor maintenance. We hypothesized that our TAZ_DEP_ and TAZ_IND_ cells could be used to identify small molecules with anti-BCSC activity because they exhibit the CSC markers and functional characteristics that are stable in a 2D culture and, hence, are amenable to HT phenotype-based screens.

**FIGURE 5:**
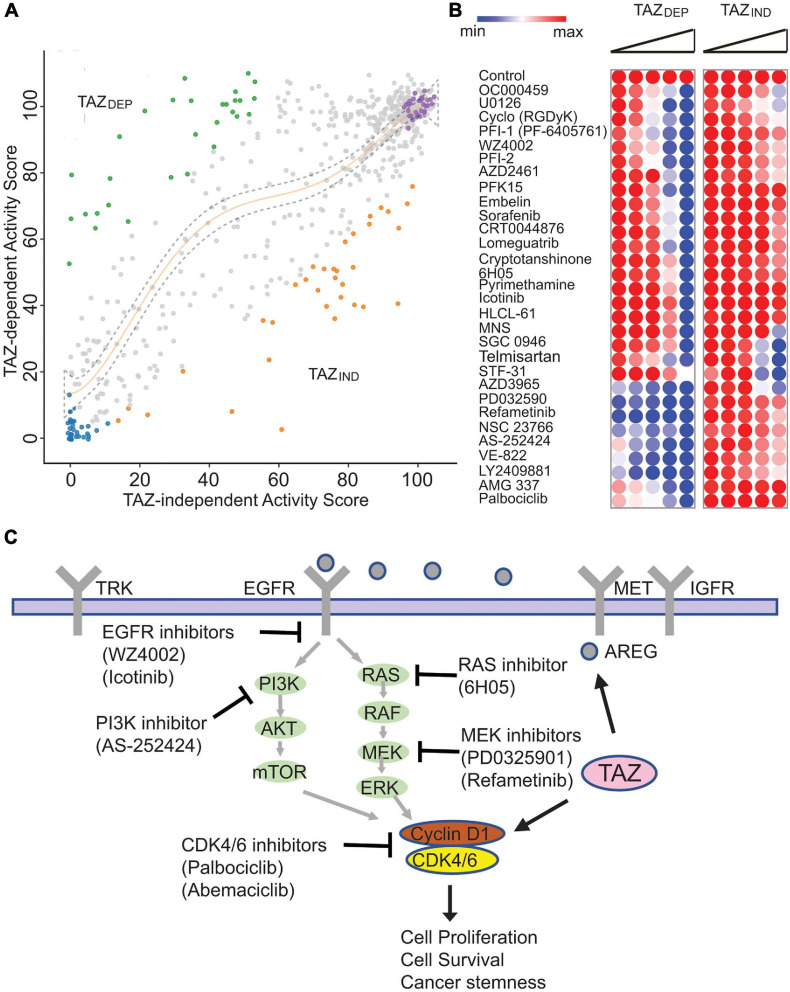
Small molecule library screen identified isogenic cell line vulnerabilities. **(A)** Results of the small molecule screen representing the relative viability of TAZ_DEP_ (y axis) and TAZ_IND_ (x axis). Polynomial regression was used to identify non-linear relationships between TAZ_DEP_ and TAZ_IND_ cells. **(B)** Heatmap of TAZ_DEP_ and TAZ_IND_ cells in response to 30 compounds. Doses from low to high are 0.65, 1.25, 2.5, 5, and 10 μM, respectively. **(C)** Summary of TAZ_DEP_ small molecule hits and their predicted MoA.

To investigate this possibility, we selected a library of ∼600 compounds that target essential cellular circuitry proteins and included FDA-approved drugs, preclinical agents, and small molecule pathway probes (Figure 5A). We annotated each compound with one or more mode of action (MoA) descriptors (Supplementary Table 3) using vendor compound catalogs, large-scale target annotation projects, and chemical databases such as DrugBank and the Therapeutic Target Database (TTD). We used this small molecule library to carry out viability screens to identify potent and selective cytostatic compounds for BC cells exhibiting a CSC-like phenotype dependent on oncogenic TAZ.

Most of the compounds had a modest effect on relative cell viability and/or growth (Figure 5A). The other hits showed a deviation from the negative controls and were categorized into three clusters: (1) hits cytotoxic to both TAZ_DEP_ and TAZ_IND_ cells; (2) hits preferentially cytotoxic to TAZ_DEP_ cells; and (3) hits preferentially cytotoxic to TAZ_IND_ cells. To identify TAZ_DEP_ cytotoxic compounds, we investigated compounds with a greater cytotoxic effect against TAZ_DEP_ vs. TAZ_IND_ cells (i.e., ratio TAZ_DEP_/TAZ_IND_ ≤ 0.75). Based on these criteria, 30 compounds showed a selective cytotoxic effect on TAZ_DEP_ cells and were selected for dose-response follow-up studies (Figure 5B).

As summarized in Figure 5C, TAZ_DEP_ cells were sensitive to most of these candidates (Supplementary Table 4). Multiple compounds were associated with known human cancer signaling pathways, including EGFR (Icotinib and WZ4002), RAS (6H050), and MEK (PD0325901 and Refametinib, and U0126-EtOH). Furthermore, we identified the small molecule inhibitors of metabolism (PFK15 and Pyrimethamine), cell cycle regulation (NSC 23766 and Palbociclib), DNA damage response (AZD2461, CRT0044876, and VE-8220), and epigenetic factors (Lomeguatrib, SGC0946, HLCL-61, and PFI-1) (Figure 5C). Notably, our chemogenomic analysis identified Cyclin D1 (CCND1)—- a well-recognized oncogene—-as a transcriptional target of TAZ. CCND1 is an activator of CDK4/6 kinases that promote the G1/S transition by inactivating RB (Malumbres and Barbacid, 2009). Palbociclib is a selective CDK4/6 inhibitor and was our top ranked hit meriting further investigation.

### Identification of CCND1 as a Direct Target Gene of TAZ

**FIGURE 6:**
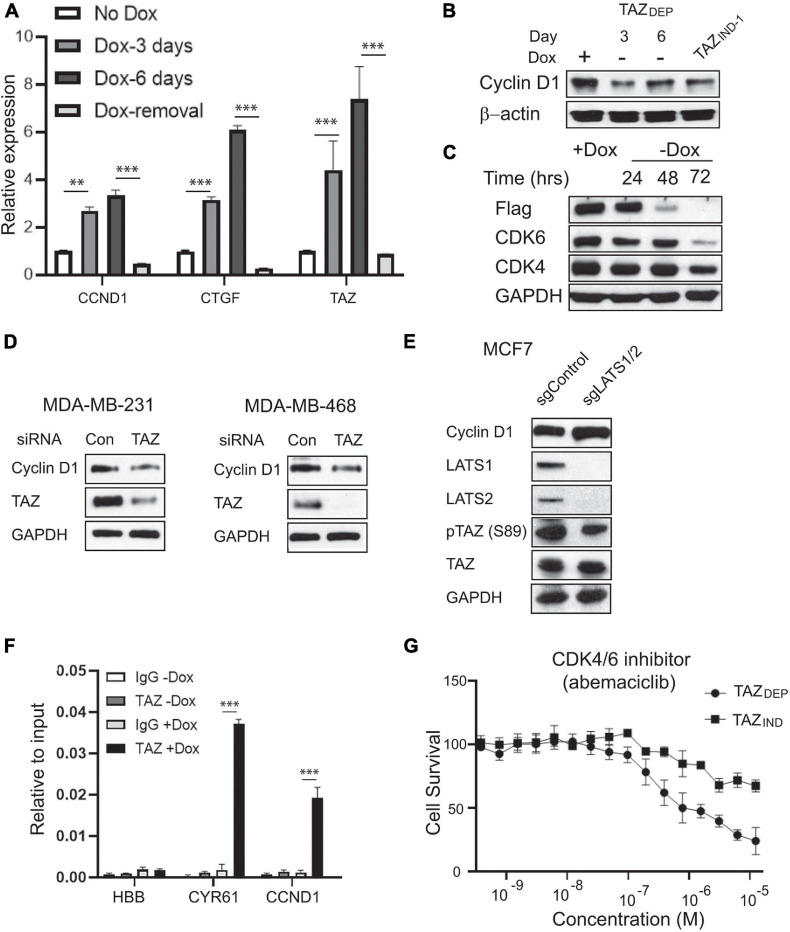
Identification of CCND1 as a direct target of TAZ. **(A)** qRT-PCR experiment detection of CCND1, CTGF, and TAZ expression in TAZ_DEP_ cells in response to dox treatment or dox removal. Relative expression was normalized by GAPDH expression. Unpaired two-tailed Student’s *t*-test: ^∗∗^*p* < 0.01; ^∗∗∗^*p* < 0.001. **(B)** Immunoblotting detection of CCND1 in response to dox withdrawal in TAZ_DEP_ cells and TAZ_IND_ cells. GAPDH was used as a loading control. **(C)** Immunoblotting detection of Flag-TAZ, CDK4, and CDK6 expression in response to dox withdrawal in TAZ_DEP_ cells. GAPDH was used as a loading control. **(D)** Immunoblotting detection of TAZ and CCND1 in siControl or siTAZ transfected MDA-MB-231 or MDA-MB-468 cells. GAPDH was used as a loading control. **(E)** Immunoblotting detection of CCND1, LATS1, LATS2, TAZ, pTAZ (S89) in sgControl or sgLATS1/2 cells. GAPDH was used as a loading control. **(F)** qPCR experiment detection of TAZ-ChIPed DNA for CCND1. CYR61 was used as positive control; HBB was used as negative control. Relative enrichment was compared with 2% of input DNA. Unpaired two-tailed Student’s *t*-test: ^∗∗∗^*p* < 0.001. **(G)** IC50 of CDK4/6 inhibitor Abemaciclib treatment. TAZ_DEP_ cells grown in the presence of 2 μg/ml dox.

The molecular mechanisms underlying TAZ-driven oncogenic transcriptional responses’ specificity remain largely unknown (Zanconato et al., 2016). Because of the limitations in discriminating functional and incidental (secondary) gene expression, it is challenging to identify the genes directly regulated by TAZ solely from transcriptome profiling using a single (static) data point. Accordingly, to confirm CCND1 is a direct target of TAZ, we treated TAZ_DEP_ cells with dox for 3 and 6 days and/or removed dox for an extra 6 days. We harvested total RNA and performed a qRT-PCR analysis. CCND1 gene expression was significantly increased in response to TAZ activation but rapidly diminished upon dox removal (Figure 6A). In addition, CCND1 protein expression decreased in response to dox removal (Figure 6B).

It has been recently reported that the loss of FAT1 led to marked elevations in CDK6 through YAP/TAZ activation (Li et al., 2018). Therefore, we quantitated CDK6 protein expression in TAZ_DEP_ cells. We found CDK6 protein levels reduced in response to dox removal (Figure 6C). To further investigate whether TAZ regulates CCND1 gene expression, we perturbed TAZ in MDA-MB-231 and MDA-MB468 BC cell lines. As shown in Figure 6D, siRNA knockdown of TAZ reduced CCND1 expression in both triple-negative BC cell lines (Figure 6D). Furthermore, we detected increased CCND1 expression in LATS1/2-null cells (Hippo pathway kinases that negatively regulate TAZ) (Ma et al., 2021; Figure 6E).

TAZ lacks an intrinsic DNA-binding domain and is thought to exert its co-activator function by binding to target promoter sequences via interactions with many different transcription factors (TFs). Among these, the TEAD family members play a dominant role as primary mediators of TAZ-dependent gene regulation and tumor-promoting activity (Zhang et al., 2009). We used the LASAGANA Search 2.0 algorithm to analyze which TFs bind to the CCND1 promoter using matrix-derived models from JASPAR and TRANSFAC databases. We found two regions with predicted TEAD tandem consensus motifs (5’-GGAATG-3’). To validate the in-silico analysis for TEAD-binding sites on the CCND1 promoter, we performed chromatin immunoprecipitation (ChIP) assays and showed TAZ directly bind to CCND1 promoter region (CYR61, a canonical transcriptional target of TAZ, was used as a positive control, and HBB served as a negative control) (Figure 6F).

The ability of D-type cyclin family members to activate CDK4 and CDK6 is the most extensively documented mechanism for their oncogenic actions (Knudsen and Witkiewicz, 2017). Selective CDK4/6 inhibitors, such as Palbociclib, Ribociclib, and Abemaciclib, have been developed. However, the *in vivo* functions of CDK4/6 inhibition are complex and extend beyond simply enforcing cytostasis. To confirm that CCND1 overexpression could be pharmacologically targeted/exploited, we investigated TAZ_DEP_ and TAZ_IND_ cell sensitivity to Abemaciclib. TAZ_DEP_ cells were more sensitive to Abemaciclib treatment than TAZ_IND_ cells (Figure 6G).

Overall, we have shown that TAZ positively regulates CCND1 expression by directly binding to its promoter region. Furthermore, the perturbation of the TAZ-CCND1-CDK4/CDK6 signaling axis led to the inhibition of TAZ_DEP_ cell proliferation, providing a rationale for its exploitation as a target in BCSC therapy.

## Discussion

The CSC model has been established as a cellular mechanism that contributes to phenotypic and functional heterogeneity in BC and other human tumors. The clinical applicability of the CSC concept to predicting patient responses remains a fundamental biomedical question. As such, the delineation of critical genes and/or pathways that can distinguish CSCs from their normal counterparts may provide novel opportunities for therapeutic intervention and help overcome tumor heterogeneity and therapeutic resistance. In the current study, we describe a simple experimental workflow that allows for the rapid isolation of BCSCs from mammary tumors with defined genotypes.

The Hippo pathway plays a critical role in cell proliferation, survival, migration, tumorigenesis, and metastasis (Yu et al., 2015). The fact that deregulated Hippo signaling is essential for a tumorigenic subpopulation with stem cell properties raises the possibility that the therapeutic activation of Hippo signaling or a pharmacological blockade of its downstream effectors could improve current cancer treatment strategies. Indeed, we hypothesize that targeting the Hippo pathway is an effective CSC therapeutic strategy. Unfortunately, the direct pharmacological inhibition of oncogenic TAZ or YAP is challenging because these proteins have no known catalytic activity or function through engaging in domains that facilitate context-dependent protein–protein interactions with diverse upstream kinases, apical–basal cell polarity proteins, or transcription factors (Harvey et al., 2013; Hansen et al., 2015). Here, we employed an alternative strategy to identify BCSC-specific druggable targets lying downstream of TAZ whose pharmacological perturbation influenced their tumor-initiating properties.

A systematic evaluation of drugs that specifically target BCSCs has been hindered because of the difficulty in isolating these cells from the bulk of tumor tissues or cell lines and the manipulations of pure populations ex vivo. Given the difficulty of targeting BCSCs therapeutically, we initially sought to characterize the transcriptional programs used by TAZ to confer CSC-related traits and predict cancer-specific vulnerabilities. Using a chemical genomics approach, we identified several candidate small molecules that target TAZ-driven cellular processes. For instance, we have previously demonstrated that amphiregulin, an EGFR ligand, is a direct target of TAZ (Yang et al., 2012). Consistent with these studies, we found TAZ_DEP_ is sensitive to the EGFR inhibitors WZ4002 and Icotinib, respectively. We also identified and validated small molecules that target the downstream effectors of multiple cancer-associated signaling pathways, such as the PI3K inhibitor AS-252424, the MEK inhibitor PD0325901, and Refametinib. Interestingly, MEK inhibitors can trigger YAP/TAZ degradation in a hippo-independent manner. We also identified multiple epigenetic targets, including PFI-1 (PF-6405761), a highly selective BET (bromodomain-containing protein) inhibitor for BRD4, which is part of the YAP/TAZ-TEAD transcriptional complex (Zanconato et al., 2018; Pobbati and Hong, 2020). Correspondingly, BRD4 inhibitors have been reported to inhibit YAP/TAZ pro-tumorigenic activity in several cells or tissue contexts and cause the regression of YAP/TAZ-addicted neoplastic lesions (Zanconato et al., 2018).

Of particular interest, we identified CCND1 and CDK6 as direct transcription targets of TAZ. CCND1 is one of the most commonly overexpressed genes in human BC and causes mammary tumors in transgenic mice (Sutherland and Musgrove, 2002). CCND1 activation promotes cell cycle progression through the phosphorylation of substrates, such as RB and transcription factors, with roles in proliferation and differentiation. CCND1 also performs additional functions to regulate gene expression in the context of local chromatin and promotes cellular migration and chromosomal instability. Notably, in our small molecule screen we found CDK4/6 inhibitors—Palbociclib and Abemaciclib—preferentially inhibited TAZ_DEP_ vs. TAZ_IND_ cell survival and proliferation. CDK4/6 inhibitors have demonstrated activity against HR^+^ and HER2^–^ BC (O’Leary et al., 2016). However, many patients exhibit primary resistance to CDK4/6 inhibition and do not derive long-term benefits from these agents. To the best of our knowledge, CDK4/CDK6 inhibitors and their MoA have not been extensively studied for TAZ-addicted tumorigenicity.

Although multiple small molecules have been identified, and further application of this method may enable the discovery of additional small molecules, some limitations merit discussion. First, all experiments were conducted using a limited set of TAZ_DEP_ and TAZ_IND_ cell lines, thereby potentially limiting the interpretability of our findings. Second, the overarching focus of our study was the discovery of TAZ_DEP_ vulnerabilities. Notably, BCSCs are phenotypically and functionally heterogeneous cells promoting tumor cell growth, progression, recurrence, and treatment resistance. As such, different oncogenic drivers will need to be investigated to determine the generality of our findings. Finally, targeting TAZ-dependence in BC cells by inhibiting CCND1-CDK4/CDK6-mediated cell cycle progression does not necessarily support the exclusive context to BCSCs and warrants further investigation.

In summary, our data reveal that TAZ-mediated tumor growth may lead to cellular plasticity and dedifferentiation. In addition, an oncogenomic analysis using pathway-specific probes identified that TAZ-expressing-driven cells are sensitive to CDK4/6 inhibitors and may be used as criteria for BC patient stratification, neoplastic growth, and anti-CSC therapy. More broadly, the ability to generate and characterize BC stem-like cells *in vitro* offers a cost-effective and scalable platform that can be perturbed with relative ease while also being compatible with high-throughput phenotype-based screens to reveal novel molecular vulnerabilities for BC therapeutics.

## Materials and Methods

### Cell Line and Cell Culture

MCF10A cells have previously been described and were authenticated by STR profiling (Shen et al., 2019). MCF10A cells were cultured in DMEM/F12 media (Corning, NY) supplemented with 5% horse serum (Invitrogen, MA), 1% Pen/Strep, 20 ng/mL EGF (ProSpec, NJ), 0.5 μg/mg hydrocortisone, 100 ng/mL cholera toxin, and 10 μg/mL insulin. TAZ_DEP_ or TAZ_IND_ tumors were harvested and minced. Small tumor tissue pieces were digested by collagenase at a temperature of 37∘C for 30 min. Single cell suspensions were grown in mammosphere growth conditions for 5 days. Sphere-forming cells were further digested trypsin and plated to 10 cm tissue culture dishes with MCF10A growth media. All cells were detected for being mycoplasma free. MDA-MB-231 and MDA-MB-468 cells were purchased from ATCC; MCF7- sgControl and sgLATS1/2 cells were kindly provided by Dr. Kun-Liang Guan (University of California San Diego). MDA-MB-231, MDA-MB-468 and MCF7 cells were cultured in DMEM supplemented with 10% fetal bovine serum and 1% Pen/Strep. All cells were cultured in a humidified atmosphere of 95% air and 5% CO_2_ at 37∘C.

### CD44^*high*^/CD24^*low*^ Cell Population Detection by Flow Cytometry Analysis

TAZ_DEP_ or TAZ_IND_ cells were dissociated by trypsin and passed through a 35 μm filter, and cell pellets were prepared by centrifugation. After washing with 1X phosphate buffered saline (PBS) containing 0.5% fetal calf serum (FBS), the cells were counted. Then, 1 × 10^6^ cells were resuspended in PBS/FBS and stained with APC antihuman CD44 and Brilliant Violet 421 antihuman CD24 antibody (Biolegend, CA) for 30 min on ice. Stained cells were washed and analyzed by flow cytometry.

### Mammosphere Formation Assay

TAZ_DEP_ or TAZ_IND_ cells were grown in serum-free DMEM/F12 10 ng/ml recombinant human basic fibroblast growth factor (bFGF; Gold Biotechnology, MO) 1:1 media (Gibco, NY) supplemented with EGF (20 ng/mL) and B27 (2%) in ultra-low attachment six-well plates (Corning). The mammospheres were allowed to grow for 5 days. Total mammospheres greater than 100 μm in diameter were counted under a microscope. Each experimental group was performed in triplicate.

### Immunoblot Analysis and Antibodies

Cells were lysed in a RIPA buffer (Boston Bio-Products, MA) in the presence of protease and phosphatase inhibitors (Thermo-Fisher Scientific, NY). Protein concentration was determined using the Bradford protein assay. Here, 20–30 ug of protein was loaded and separated by SDS-PAGE; then, it was transferred onto PVDF membranes (EMD Millipore, MA). Membranes were blocked in 5% milk in TBS-T for 1 h at room temperature and incubated overnight at 4∘C with primary antibodies. The membranes were washed and incubated with HRP-congregated antimouse or antirabbit secondary antibody (Bio-Rad, CA) for 1 h at room temperature. Proteins were detected using the ECL Western blotting substrate (Thermo-Fisher Scientific, NY). CDK4, CDK6, TAZ, YAP, E-cadherin, and vimentin antibodies were purchased from Cell Signaling Technology; Fibronectin antibody from BD Biosciences; Flag (M2) antibodies from Sigma-Aldrich; and anti-GAPDH from Ubiquitin-Proteasome Biotechnologies.

### RNA Extraction and Quantitative Real-Time PCR

Total RNA was harvested using Trizol Reagent (Life Technologies, NY) according to the manufacturer’s instructions. cDNA synthesis and quantitative real-time PCR were performed. GAPDH was used as the internal control. The primer sequences were as follows:

TAZ-F: 5′-AGTACCCTGAGCCAGCAGAA-3′;

TAZ-R: 5′-GATTCTCTGAAGCCGCAGTT-3′;

CTGF-F: 5′-GGAAATGCTGCGAGGAGTGG-3′;

CTGF-R: 5′-GAACAGGCGCTCCACTCTGTG-3′;

CYR61-F: 5′-CACACCAAGGGGCTGGAATG-3′

CYR61-R: 5′-CCCGTTTTGGTAGATTCTGG-3′

CCND1-F: 5′-GTTCGTGGCCTCTAAGATGAAG-3′

CCND1-R: 5′-GATGGAGTTGTCGGTGTAGATG-3′

PTPN14-F: 5′-GGAAGTTGCAGAGGTAGATAGTG-3′

PTPN14-R: 5′-GGGAAAGGACAGCAGCTAAA-3′

ANKRD1-F: 5′-AGTAGAGGAACTGGTCACTGG-3′

ANKRD1-R: 5′-TGGGCTAGAAGTGTCTTCAGAT-3′

CRIM1-F: 5′-GCCCAGTGTGGTGAGATAAA-3′

CRIM 1-R: 5′-GCAGCCAGCGGGATTATTA-3′

NT5E-F: 5′-GGAGATGGGTTCCAGATGATAAA-3′

NT5E-R: 5′-CGACCTTCAACTGCTGGATAA-3′

GAPDH-F: 5′-GTGAAGGTCGGAGTCAACGG-3′

GAPDH-R: 5′-GAGGTCAATGAAGGGGTCATTG-3′

AXL-F: 5′-GTC CTC ATC TTG GCT CTC TTC-3′

AXL-R: 5′-GAC TAC CAG TTC ACC TCT TTC C-3′

### Cell Proliferation, Cell Survival, Colony Formation

Three thousand cells were plated into 96-well plates with or without 2 μg/ml dox. The plates were harvested daily for 7 days. Ten microliters of resazurin (R&D Systems, MN) were added to each well and incubated for 4 h at 37°C. Fluorescence was read using 544 nm excitation and 590 nm emission wavelengths. The cell proliferation rate was calculated using the first day fluorescence read as the baseline.

For the drug treatment assay, the cells were plated into a 96-well plate. The next day, serial diluted (1:2; start with 20 μm) drugs were added and incubated at 37°C for 72 h. Resazurin assay was performed. Cisplatin, Paclitaxel, Salinomycin, and Abemaciclib were purchased from Tocris (MN).

For colony formation assay, cells were trypsin and counted. Approximately 200 cells/well were plated into a six-well plate and grown in present or absent of 2 μg/ml dox in 37°C about 8–10 days. Cells were washed with PBS and stained with 10% crystal violate for 2 h at room temperature. Images were taken, and colonies were counted. Each accumulation of more than 50 cells was counted as a positive colony. Each sample was performed in triplicate, and three independent experiments were performed.

For colony formation in soft agar, 0.5% agar containing cell grow media was plated into a six-well plate. 5 × 10^4^ cells were suspended in a growth medium mixed with 0.4% agar and seeded into a base agar containing a six-well plate. Cells were incubated in the presence of or without 2 μg/ml dox in 37°C for 2 weeks. Colonies were stained with 0.02% iodonitrotetrazolium chloride (Sigma-Aldrich, MO) and photographed. Colonies larger than 50 μm in diameter were counted as positive for growth. Assays were conducted in triplicate in three independent experiments.

### 3D Morphogenesis and Mammosphere Formation

Four thousand cells were cultured in growth factor–reduced reconstituted basement membrane (Matrigel; BD Biosciences) in an eight-well Nunc^TM^ Lab-Tek^TM^ II chamber slide (Thermo-Fisher Scientific, NY) as described previously (Debnath et al., 2003). Cells were grown in the presence of or without 2 μg/ml dox in 37°C for 10 days. The cell lines were assayed in three independent experiments.

### Transwell Migration Assays

Transwell migration assays were performed as previously described (Mussell et al., 2020). Briefly, 5 × 10^4^ cells were plated on Transwell inserts (8 μm pore size; Corning, NY) in assay medium at 37°C for 24 h. The Transwell inserts were washed with PBS and wiped with a Q-Tip, fixed with 4% paraformaldehyde, and stained with 10% crystal violet for 2 h at room temperature. Migrated cell numbers were counted, and assays were conducted in duplicate in three independent experiments.

### *In vivo* Tumor Growth and Immunohistochemistry

To start, 1 × 10^6^ TAZ_DEP_ or TAZ_IND_ cells were injected into the mammary fat pad of 6–8-week-old female SCID mice. For serial dilution experiments, 1 × 10^6^, 1 × 10^5^, or 1 × 10^4^ TAZ_DEP_ or TAZ_IND_ cells were injected into the mammary fat pads of 6–8-week-old female SCID mice. The SCID mice were bred at the Roswell Park Comprehensive Cancer Center (RPCCC). Tumor sizes were measured once a week using a caliper. Mammary tumor formations were also detected by the *In Vivo* Luminescence Imaging System. The care and use of the animals were approved by the Institutional Animal Care and Use Committee of the RPCCC (Buffalo, NY).

For immunohistochemistry (IHC) staining, formalin-fixed paraffin embedded (FFPE) tissue blocks were sectioned 5 microns thick and subjected to hematoxylin and eosin (H&E) and IHC staining. The quality of the histomorphology of tumor samples was assessed by H&E staining. Antibodies against Ki-67 was obtained from Dako. Histomorphology and immunostaining results were interpreted by a board-certified pathologist (BX).

### Small Molecule Library Screen and Validation

The small molecule library was purchased from Selleckchem (TX). Screening was performed by the Small Molecule Screen Shared Resource at RPCCC. TAZ_DEP_ or TAZ_IND_ cells were plated into 384-well plates and treated with 10 μM inhibitors 24 h later in duplicate. Resazurin (Sigma-Aldrich, MO) cell enumeration assay was performed after 72 h. Cell survival for each drug was compared with that of DMSO-treated controls.

For drug validation, 30 selected small molecules were serially diluted and dispensed with automated reagent dispensers into 384-well plates. Cell survival was measured after 72 h by resazurin assay.

### RNA-Seq and ChIP Assay

For the RNA-seq analysis, RNA was extracted from 60% confluent monolayers of cells, as described above. The RNA samples were subjected to transcriptome sequencing (RNA-seq) with an Illumina HiSeq 2000 sequencer in the RPCCC genomic shared resource. Raw reads passed quality filter from Illumina RTA were mapped to the mm10 mouse reference genomes and corresponding GENCODE (v12) annotation databases using STAR two-pass algorithm (Dobin and Gingeras, 2015). The mapped bam files were further QCed using RSeQC (Wang et al., 2012), a quality control Bioconductor R package for RNASeq data, to identify potential RNASeq library preparation problems. From the mapping results, the read counts for genes were obtained by featureCounts from Subread (Liao et al., 2013). Transcript level quantification were generated using kallisto (Bray et al., 2016), an alignment free tool. Dara normalization and differentially expression analysis were performed using DESeq2 (Love et al., 2014), a variance-analysis package developed to infer the statically significant difference in RNA-seq data. Pathway analysis was done by GSEA (Subramanian et al., 2005) pre-ranked mode using ranked gene list based on test statistics from DE analysis against the hallmark (H) and the canonical pathways in MSigDB. The volcano plots were generated using Enhanced Volcano Bioconductor package and the heatmaps were generated using heatmap R package.

For the ChIP analysis, chromatin immunoprecipitation assays were performed using the SimpleChIP Enzymatic Chromatin IP Kit (Magnetic Beads; Cell Signaling Technology, MA). Briefly, inducible TAZ-expressing MCF10A cells were given or withheld from dox treatment for 72 h. Cells were cross-linked, lysed, and sonicated to generate DNA fragments with an average size of 0.5 kb. Immunoprecipitation was performed using 5 μg antibody to IgG or TAZ (catalog number #4883; Cell Signaling Technology, MA), respectively. ChIPed DNA was subjected to real-time PCR.

The primer sequences were as follows:

CYR61-F: 5′-CACACACAAAGGTGCAATGGAG-3′

CYR61-R: 5′-CCGGAGCCCGCCTTTTATAC-3′

HBB-F: 5′-GCTTCTGACACAACTGTGTTCACTAGC-3′

HBB-R: 5′-CACCAACTTCATCCACGTTCACC-3′

CCND1-F: 5′-AAC TCG CTG GGC AAG TC-3′

CCND1-R: 5′-TAG GGA ATT CTG GGT CCT CA-3′

### RNAi Assay

A mixture of four siRNAs (SMARTpool) targeting TAZ and non-targeting (control SMARTpool) siRNA were purchased from Dharmacon. RNAi transfection was performed according to the manufacturer’s instructions. Cell lysate was harvested after 72 h RNAi transfection and followed by immunoblot.

### Statistical Analysis

All statistical analyses of cell proliferation, cell migration, colony formation, FACS analysis, mammosphere formation, RT-PCR, and soft agar assay were performed with two-tailed Student’s *t*-tests; data are expressed as mean ± SD.

## Data Availability Statement

The data presented in the study are deposited in the Gene Expression Omnibus (GEO) repository, accession number is: GSE168672.

## Ethics Statement

The animal study was reviewed and approved by Institutional Animal Care and Use Committee of the RPCCC (Buffalo, NY).

## Author Contributions

CF and JZ designed the study and wrote the manuscript. HS, YC, YW, JW, YZ, LW, QH, TL, BX, and MC performed the experiments and data analysis. All authors contributed to the article and approved the submitted version.

## Conflict of Interest

CF was a consultant/advisory board member for Cellecta, Inc (Mountain View, CA). The remaining authors declare that the research was conducted in the absence of any commercial or financial relationships that could be construed as a potential conflict of interest.
