# Supplementary material for: Identification of TAZ-Dependent Breast Cancer Vulnerabilities Using a Chemical Genomics Screening Approach
Source: Front Cell Dev Biol. 2021 Jun 15;9:673374. doi: 10.3389/fcell.2021.673374 (PMC8239392; doi:10.3389/fcell.2021.673374)
Supplement: Supplementary file 5 [file Data_Sheet_1.pdf]

Identification of TAZ-dependent breast cancer vulnerabilities using a chemical genomics screening approach

He Shen[1], Yanmin Chen[1], Yin Wan[1], Tao Liu[2], Jianmin Wang[2], Yali Zhang[2], Lei Wei[2], Qiang Hu[2], Bo Xu[3], Mikhail Chernov[4], Costa Frangou[5*] and Jianmin Zhang[1*]

## *Supplemental Material*

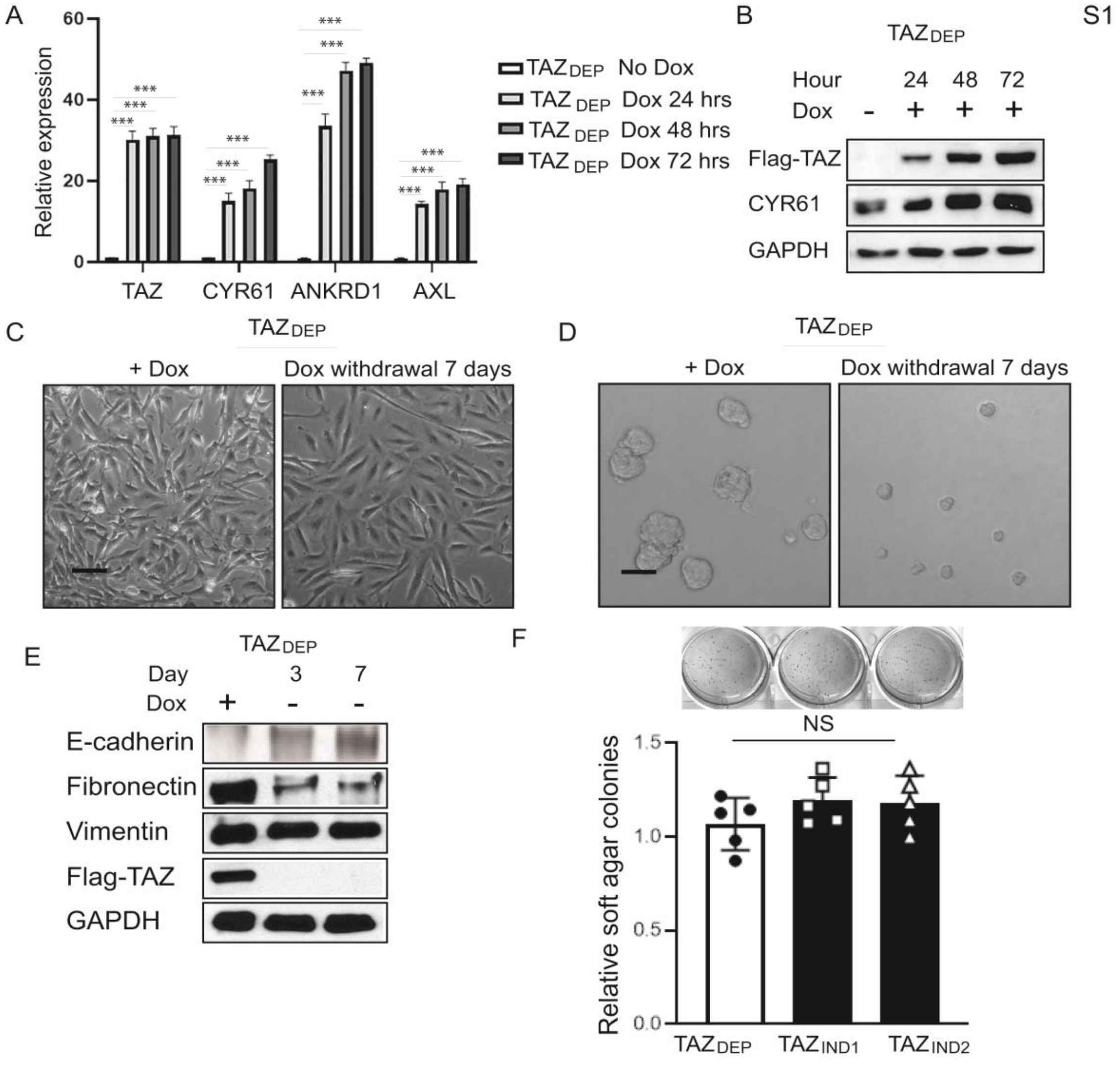


**Figure S1 Dox withdrawal inhibits $TAZ_{DEP}$ cell proliferation**

**A.** qRT-PCR experiment detection of TAZ, CYR61, ANKRD1 and AXL expression in $TAZ_{DEP}$ cells in response to dox treatment. Relative expression was normalized by GAPDH expression. Unpaired two-tailed student t-test: *** p<0.001

B. Immunoblotting detection of CYR61 in response to dox treatment in $TAZ_{DEP}$ cells. GAPDH was used as a loading control.

C. Representative images of $TAZ_{DEP}$ cell morphology in a 2D culture under the condition of Dox treatment or Dox withdrawal. $TAZ_{DEP}$ cells grown in the presence of 2 µg/ml dox. Scale bar=50 µm.
D. Representative images of $TAZ_{DEP}$ cell morphology in a 3D culture under the condition of Dox treatment or Dox withdrawal. $TAZ_{DEP}$ cells grown in the presence of 2 µg/ml dox. Scale bar=50 µm.
E. Representative images and quantification of colony formation in soft agar of $TAZ_{DEP}$ and $TAZ_{IND}$ cells. Data are shown as the mean ± SD. Unpaired two-tailed student t-test: NS=not significant.

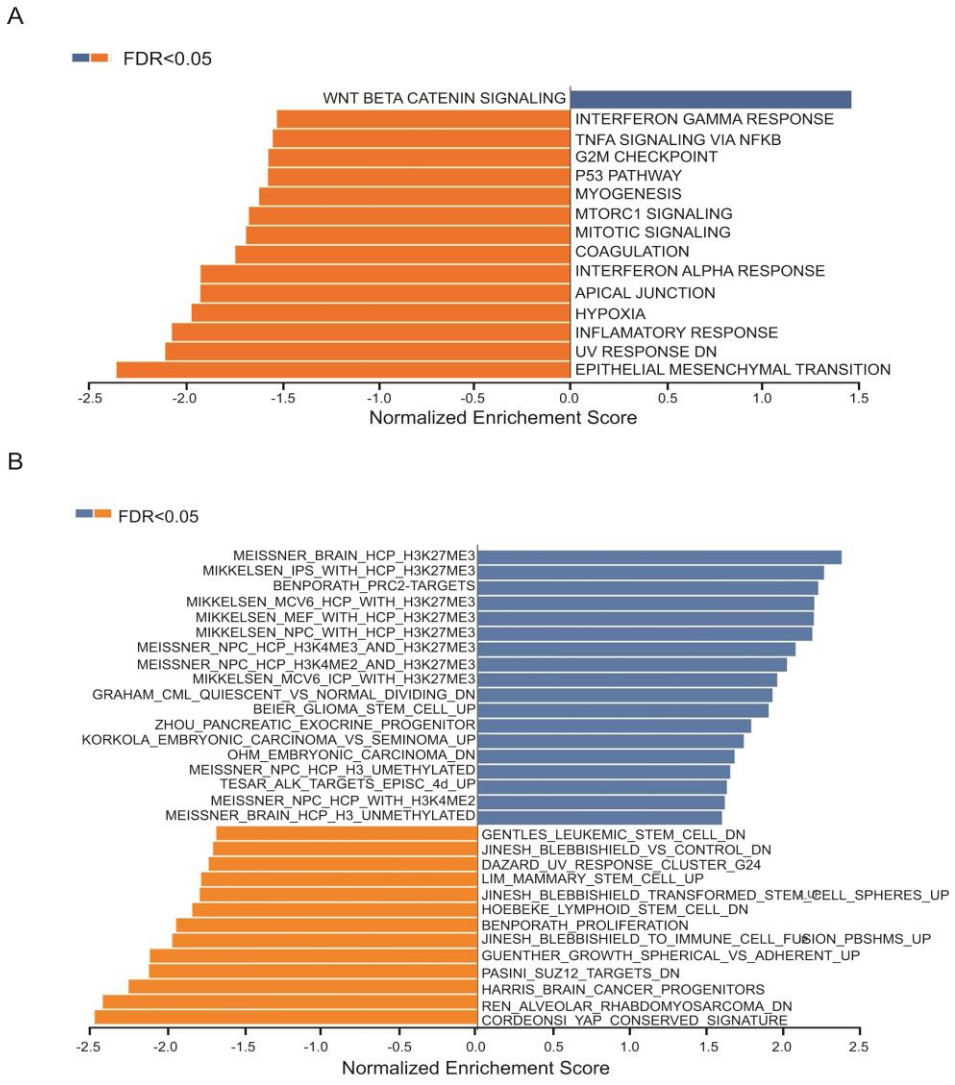


**Figure S2 KEGG and GO term analysis of $TAZ_{DEP}$ and $TAZ_{IND}$ enriched pathways.**
A. KEGG analysis for pathway alterations in $TAZ_{DEP}$ and $TAZ_{IND}$ cells.
B. GO term analysis identified enrichment biological processes in $TAZ_{DEP}$ and $TAZ_{IND}$ cells.



This is a provisional file, not the final typeset article

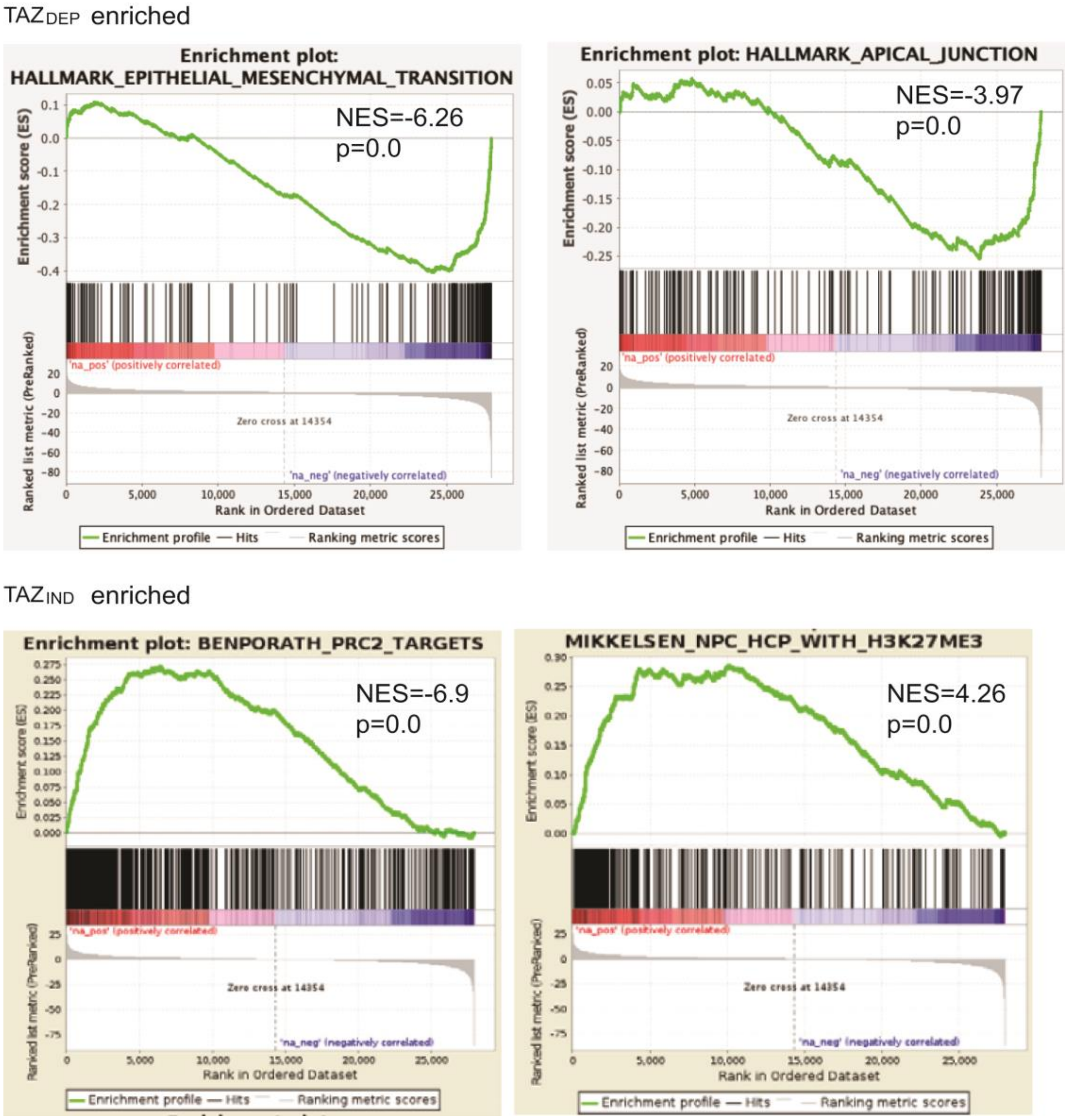


**Figure S3 GSEA analysis of TAZ$_{DEP}$ and TAZ$_{IND}$ enriched pathways.**

## Supplemental Tables

**Table-S1 Genetically characterization of TAZ$_{DEP}$ and TAZ$_{IND}$ cells.**

**Table-S2 Differential expression gene changes of TAZ$_{IND}$ versus TAZ$_{DEP}$ cells.**

**Table-S3 List of ~600 compounds library.**

**Table-S4 List of $TAZ_{DEP}$ cells sensitive compounds.**



This is a provisional file, not the final typeset article
